# Egg yolk antibody combined with bismuth-based quadruple therapy in *Helicobacter pylori* infection rescue treatment: a single-center, randomized, controlled study

**DOI:** 10.3389/fmicb.2023.1150129

**Published:** 2023-05-16

**Authors:** Sha Cheng, Huan Li, Ju Luo, Jingshu Chi, Wenfang Zhao, Jiahui Lin, Canxia Xu

**Affiliations:** Department of Gastroenterology, The Third Xiangya Hospital of Central South University, Changsha, Hunan, China

**Keywords:** *Helicobacter pylori*, egg yolk antibody (Ig Y), Ig Y-*H. pylori*, rescue therapy, bismuth-based quadruple therapy

## Abstract

**Background:**

The increasing antibiotic resistance is the main issue causing *Helicobacter pylori* (*H. pylori*) eradication failure. As a nutritional supplement, Egg Yolk Antibody (Ig Y) provides a new approach for *H. pylori* infection rescue therapy.

**Methods:**

In this randomized, controlled study, 100 *H. pylori*-positive patients with previous *H. pylori* eradication treatment were included. All individuals received standard bismuth-containing quadruple therapy twice daily (5 mg ilaprazole, 100 mg doxycycline, 500 mg clarithromycin or 1 g amoxicillin or 100 mg furazolidone, and 220 mg colloidal bismuth tartrate) for 14 days and were randomized to receive either twice daily 7 g Ig Y-*H. pylori* treatment (study group) or not (control group). 4 weeks after the end of treatment, urea breath tests were used to assess the *H. pylori* eradication rate. All participants scored by the Global Overall Symptom scale (GOS) and recorded adverse events during the trial.

**Results:**

The *H. pylori* eradication rates were 84.0% (95% CI 73.5–94.5%) vs. 80.0% (95% CI 68.5–91.5%) in the study and control groups at intention-to-treat (ITT) analysis and 85.7% (95% CI 75.6–95.9%) vs. 80.0% (95% CI 68.5–91.5%) at per-protocol (PP) analysis, respectively. The number of over 80% symptom relief after treatment in the two groups was 27 (60%) and 12 (29.2%) (*p* < 0.05), and the incidences of adverse events were 4 (8%) and 6 (12%), respectively.

**Conclusion:**

Both groups achieved satisfactory eradication efficiency in *H. pylori* rescue therapy and Ig Y-*H. pylori* effectively alleviates the symptoms with good compliance and fewer adverse effects.

## Introduction

*Helicobacter pylori* (*H. pylori*) was recognized as a class I carcinogen by the World Health Organization in 1994 and ranked as a high-priority bacterium in the list of the deadliest superbugs threatening human health in 2017 (Dang and Graham, 2017; Mommersteeg et al., 2018; Tacconelli et al., 2018). Although the overall global prevalence of *H. pylori* infection is declining, the consequences should not be underestimated (Ansari and Yamaoka, 2022; Wang et al., 2022).

Increased antimicrobial resistance in *H. pylori* contributes to the declined efficiency of previous standard eradication, especially in rescue treatments (Han et al., 2022). Available studies have shown that the eradication rate of standard triple therapy is already below 80% (Savoldi et al., 2018). Other improved regimens, including bismuth-containing quadruple therapy, concomitant therapy, and sequential therapy, to some extent help to enhance eradicating effectiveness. However, about 3 to 24% of patients still failed at first-line therapy (Lin and Hsu, 2018). Clearly, more appropriate regimens are required for rescue therapies (Peitz et al., 2002). Most guidelines recommend low-resistance antibiotic regimens, such as levofloxacin-based therapy, as a second-line treatment for *H. pylori* infection. Still, some clinical studies noted that these regimens failed to demonstrate satisfactory eradication rates and adverse effects (Bilardi et al., 2004; Gisbert et al., 2007). Hence, in the context of high global antibiotic resistance, it is essential to explore novel and remedial approaches to reduce multiple treatments failure of *H. pylori*.

Egg Yolk Antibody (Ig Y), also known as yolk immunoglobulin Y, is a particular immunoglobulin produced by specific pathogens-activated avian B lymphocytes. It is transferred from serum to yolk, then used to treat relevant pathogens by passive immunization. It is worth pointing out that Ig Y is a normal human dietary component, which is safe and free of drug resistance (Müller et al., 2015). The Food and Drug Administration (FDA) has classified yolk antibodies in the “generally recognized as safe” category. Previous studies indicated that Ig Y was considered as a potential antibiotic alternative for oral, respiratory and gastrointestinal infections (Rahman et al., 2013; Abbas et al., 2019; El-Kafrawy et al., 2023). Furthermore, Ig Y is structurally similar to mammalian immunoglobulin G (IgG) but does not bind human Fc receptors or complement system (Fink et al., 2017; Zhang et al., 2021). Compared to the traditional method of obtaining antibodies from animal serum, stable Ig Y can be extracted in large quantities from egg yolk, a non-invasive preparation process that is more economical and ethical (Mine and Kovacs-Nolan, 2002; Kovacs-Nolan and Mine, 2012). Attallah et al. (2009) found that yolk antibodies (IgY-Hp58) obtained from *H. pylori*-stimulated immunization effectively inhibited *H. pylori* infection and alleviated gastritis in BALB/c mice. Another clinical study showed that oral anti-Hp mIgY for 2 weeks reduces urea breath test values and inhibits *H. pylori* activity (Guo et al., 2021). Currently, Ig Y is used as an oral passive immunotherapy for preventing and controlling gastric and oral infections, with active applications in treating viral diarrhea and other gastrointestinal diseases. However, most existing Ig Y-*H. pylori-*related studies have been conducted in animal studies (Nomura et al., 2005; Yang et al., 2012; Hong et al., 2018), and fewer clinical studies. Thus, this single-center, randomized, controlled study aimed to find novel, safe and effective approaches for patients who have failed *H. pylori* eradication therapy, and to identify the feasibility of Ig Y-*H. pylori* as a supplementary treatment to rescue therapy.

## Materials and methods

### Study design and patients

This single-center, prospective, randomized controlled trial was conducted in The Third Xiangya Hospital of Central South University between April 2022 and December 2022. Subjects were randomly assigned to study or control group based on randomized tables generated by random number generators. The study was approved by the committee of central research institution (Third Xiangya Hospital of Central South University) and obtained the informed consent from the participants.

Adult *H. pylori*-infected subjects who had taken complete standard eradication therapies but failed were recruited. *H. pylori* infection was determined by 13C/14C urea breath test (UBT) or/and rapid urease test, or/and histological testing of gastric mucosa. None of the participants were taking H2 receptor antagonists or proton pump inhibitors (PPIs) within the past 2 weeks, or antibiotics, bismuth or any other medications that might affect *H. pylori* activity within the past 4 weeks. Exclusion criteria were as follows: (1) allergy to Ig Y product components or drugs used in this study; (2) recent history of gastrointestinal hemorrhage, obstruction, perforation, tumor, and other gastrointestinal severe diseases; (3) other out gastrointestinal severe diseases; (4) mental illness, psychological disorders that cannot be expressed commonly; (5) pregnancy or lactation.

### Interventions

The eligible subjects were randomly divided into the study or control group based on a computerized random sequence. The control group was treated with bismuth-containing quadruple therapy (5 mg ilaprazole, 100 mg doxycycline, 500 mg clarithromycin or 1 g amoxicillin or 100 mg furazolidone, and 220 mg colloidal bismuth tartrate) twice daily for 14 days. The study group received an additional Ig Y-*H. pylori* (7 g, twice daily) for 14 days. We avoided reusing patients’ previous antibiotics empirically. Detailed medications were administered as shown in the Table 1.

**TABLE 1:** Drug use of two therapeutic regimens.

Study group	Control group	Drugs	Doses	Course
√	√	Ilaprazole	5 mg, Bid	14 Days
√	√	Doxycycline	100 mg, Bid	14 Days
√	√	Clarithromycin/ amoxicillin /furazolidone	500 mg, Bid/1 g, Bid/100 mg, Bid	14 Days
√	√	Colloidal bismuth tartrate	220 mg, Bid	14 Days
√		Ig Y-*H. pylori*	7 g, Bid	14 Days

**FIGURE 1:**
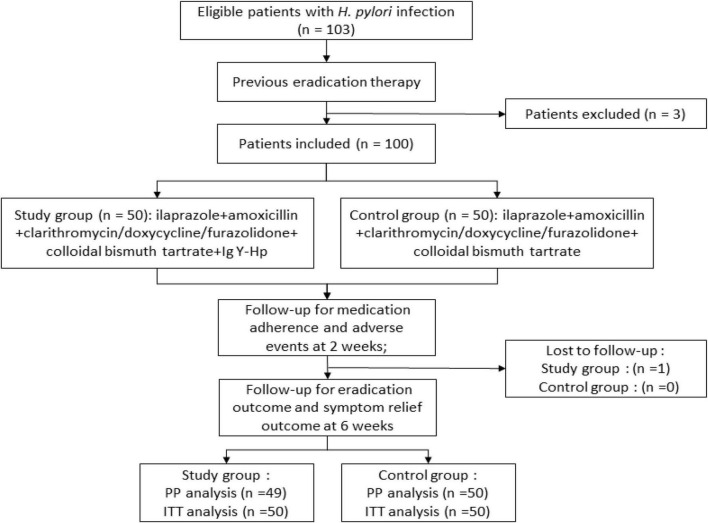
Flow diagram of the study. Hp, *H. pylori*.

The entire study design is shown in Figure 1. Before receiving treatment, all subjects received face-to-face training and were given written cards to record drugs use, adverse effects, and clinical symptoms. Satisfactory adherence was defined as taking more than 80% of the total medication.

### Follow-up and assessment of study endpoints

Subjects were followed up three times after being screened and enrolled (day 0). The 1st visit after 2 weeks of medication (day 14 ± 2), the 2nd visit after 4 weeks of medication (day 28 ± 2), and the review after 6 weeks of medication, along with the last 3rd visit (day 42 ± 2). During *H. pylori* eradicating treatment, patients took their medication followed by prescription and counted the remaining at the end of the quantity. Patients were encouraged to contact the investigator in case of discomfort or doubt to gain good compliance and safety.

### Outcomes

The primary outcome of the trial was the *H. pylori* eradication rate (number of successful eradications/number of eradications), which was diagnostic by 13/14C-UBT. One of the secondary outcomes was symptoms remission, where subjects’ gastrointestinal symptoms were scored by the 7-point Global Overall Symptom scale (GOS) before and after treatment, respectively, and scored as cured (≥80% decrease in score after treatment), remission (0–80% decrease in score after treatment), and ineffective (no reduction in score or worsening of symptoms after treatment) (Veldhuyzen van Zanten et al., 2006). The other secondary outcome was the incidence of adverse events.

### Statistical analysis

The data were analyzed using SPSS.26.0 software. Categorical variables were described by frequencies and proportions (%), and continuous variables were described by mean and standard deviation (SD), unless otherwise stated. The *H. pylori* eradication rates were calculated by per-protocol (PP) and intention-to-treat (ITT) analysis, expressed as percentages, and assessed with 95% confidence intervals. Significance of categorical data was assessed using the chi-square test or Fisher’s exact test, while continuous variables were tested using the *t*-test. *p* < 0.05 represents statistical significance.


$$\mathrm{Eradication}\,\mathrm{rate}\,\left(\mathrm{PP}\right)=\frac{\mathrm{Number}\,\mathrm{of}\,\mathrm{eradication}\,\mathrm{successes}}{T\,o\,t\,a\,l\,n\,u\,m\,b\,e\,r\;-\;C\,a\,s\,e\,d\,r\,o\,p\,p\,e\,d},$$

$$\mathrm{Eradication}\,\mathrm{rate}\,\left(\mathrm{ITT}\right)=\frac{\mathrm{Number}\,\mathrm{of}\,\mathrm{eradication}\,\mathrm{successes}}{T\,o\,t\,a\,l\,n\,u\,m\,b\,e\,r}.$$

## Results

### Characteristics of the patients

A total of 103 patients were screened for eligibility and 100 patients (3 were excluded for refusing to participate) were randomly divided into study group (*n* = 50) and control group (*n* = 50). Patient characteristics were similar between the two groups (*p* > 0.05) (Table 2). Among all participants, 49 and 50 patients completed the entire trial and follow-up, respectively.

**TABLE 2:** General demographic data of patients.

Variables	Study group (*n* = 50)	Control group (*n* = 50)	*P*-value
Gender (male/female)	19/31 (38/62%)	22/28 (44/56%)	0.542^a^
Age (years)	46.7 ± 9.2	46.2 ± 12.7	0.829^b^
Body mass index (BMI = Kg/m2)	23.1 ± 3.3	22.9 ± 3.3	0.762^b^
Education level (high school and above)	36 (72%)	31 (62%)	0.288^a^
Cigarette smoking	7 (14%)	7 (14%)	1.000^a^
Alcohol consumption	12 (24%)	16 (32%)	0.373^a^
Dietary habit (bland diet)	21 (42%)	22 (44%)	0.771^a^
Eating customs (serving chopsticks)	22 (44%)	19 (38%)	0.542^a^
Family infection	11 (22%)	16 (32%)	0.188^a^
Symptom	45 (90%)	41 (82%)	0.249^a^
Gastroscopy			0.136^a^
Non-atrophic gastritis	33 (66%)	40 (80%)	
Atrophic gastritis	16 (32%)	8 16%)	
Polyps	8 (16%)	4 (8%)	
Peptic ulcer	7 (14%)	11 (22%)	

^a^Chi-square test; ^b^*t*-test; Data are presented as mean ± SD or number.

### *H. pylori* eradication rates

**FIGURE 2:**
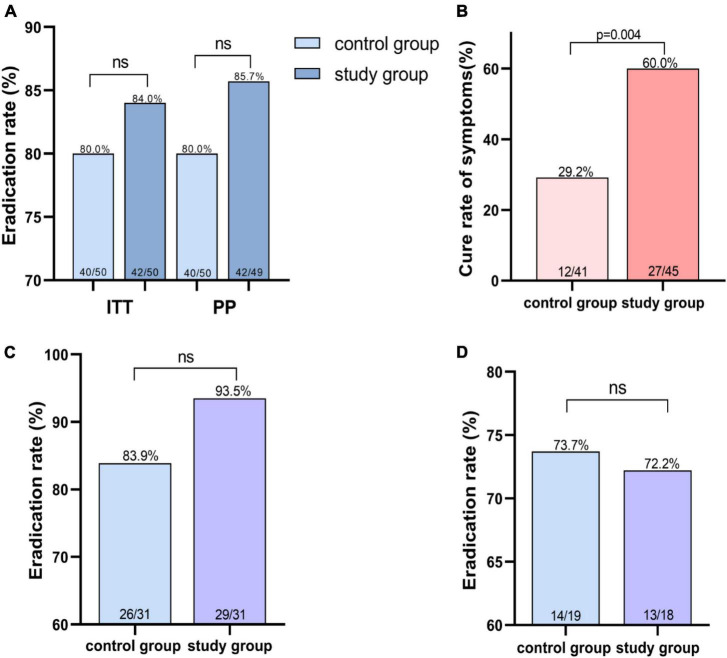
*Helicobacter pylori* eradication rate and symptom cure rate. **(A)** Eradication rate of *H. pylori* infection in different treatment groups. **(B)** Higher symptom cure rates in study group using Ig Y-*H. pylori*. **(C)** Eradication rate of second-line therapy in different treatment groups. **(D)** Eradication rates for the third or more treatments in the different treatment groups. ns indicates not significantly different.

In the ITT analysis, the eradication rates were 84% (95% CI 73.5–94.5%) in study group and 80% (95% CI 68.5–91.5%) in control group and 85.7% (95% CI 75.6–95.9%) and 80% (95% CI 68.5–91.5%) in the PP analysis (Figure 2A and Supplementary Table 3). Further subgroup analysis was performed according to repeated treatment times. The data revealed that in the population that once failed before, the eradication rates in the two groups were 93.5 and 83.9%, while in ≥2 eradication attempts, 72.2 and 73.7%, respectively (Figures 2C, D and Supplementary Table 4).

### Symptom relief

The severity of the following symptoms–upper abdominal pain, bloating (or postprandial fullness), upper abdominal discomfort, belching, heartburn, nausea, acid reflux, and early satiety–were assessed in the subjects using the Global Overall Symptom scale (Veldhuyzen van Zanten et al., 2006), and the relevant clinical symptoms were explained as shown in the Supplementary Tables 1, 2. And 45 people (study group) and 41 people (control group) showed gastrointestinal symptoms, such as abdominal pain and bloating and epigastric discomfort, and so on. The number of patients with cured, relieved and ineffective symptoms in the two groups after treatment were 27, 17, 1 (study group) and 12, 27, 12 (control group), respectively. The use of Ig Y can better ameliorate the patient’s symptoms (Figure 2B and Supplementary Table 5).

### Adverse events

**FIGURE 3:**
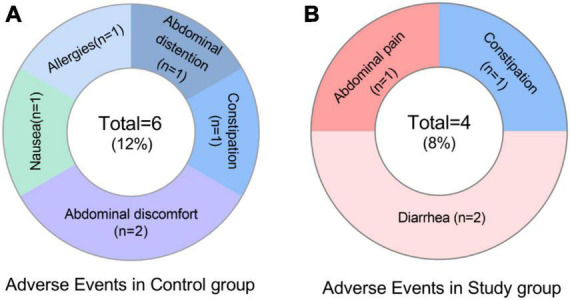
Adverse events.

The incidence of AE was 8% (4/49) and 12% (6/50) in the study and control groups, respectively. Major manifestations were abdominal pain, diarrhea, constipation, allergy (rash), bloating, abdominal discomfort and nausea (Figure 3 and Supplementary Table 6). Side effects are mainly mild or moderate and recovered after discontinuation of the drugs.

## Discussion

This study is the first randomized controlled clinical trial to investigate the effect of Ig Y-*H. pylori* as an adjuvant therapy in *H. pylori* rescue eradication treatments. Our results showed that Ig Y-*H. pylori* supplementation to the quadruple therapy significantly increased symptoms relief.

Both groups achieved good eradication rate of *H. pylori.* However, the eradication rate of the combination of Ig Y-*H. pylori* were superior to the quadruple regimen alone, using ITT (84 vs. 80%) and PP analysis (85.7 vs. 80%). After classification according to the number of eradications, we found that Ig Y- *H. pylori* increased eradication rates in second-line therapy (93.5 vs. 83.9%), but in patients with refractory *H. pylori* infection (at least 2 failed eradication), the two groups had similar eradication rates (72.2 vs. 73.7%, Figure 2 and Supplementary Table 4). Although there was no statistical difference in eradication rates between the two regimens, this may be related to the small sample size. In addition, Ig Y treated study group significantly relieved gastrointestinal symptoms (*p* < 0.05) and fewer adverse events. These results suggest that the combination Ig Y-*H. pylori* regimen can achieve high eradication efficiency and further deliver better symptom relief benefits, improving patients’ quality of life. Hence, it is a better rescue therapy option for the *H. pylori* eradication failure population.

The American Gastroenterological Association (AGA) clinical guidelines state that patients with a history of eradication failure should be treated cautiously and avoid repeat antibiotic therapy (Chey et al., 2017). The Maastricht VI/Florence consensus report recommends that fluoroquinolone-containing triple therapy regimens be considered for rescue therapy following failure of standard triple therapy, non-bismuth quadruple therapy, or bismuth quadruple therapy (Malfertheiner et al., 2022). However, some clinical studies indicate that fluoroquinolone-containing triple therapy have failed to achieve satisfactory eradication (Bilardi et al., 2004; Gisbert et al., 2007). A randomized controlled study reported 22 and 11.5% overall adverse events in a quadruple regimen containing levofloxacin with bismuth and a standard levofloxacin triple regimen for remedial treatment, respectively, with the latter having an eradication rate of only 69.2% (Hsu et al., 2017). Similarly, botanical components or probiotic adjuvant therapy for *H. pylori* can reduce drug side effects and improve adherence (Yang et al., 2021; Guo et al., 2022; Viazis et al., 2022). Other studies showed that individualized and precise treatment based on drug sensitivity testing is another better rescue therapy choice, but the high cost of gastroscopy and drug sensitivity testing brings higher economic costs and invasive injury, even as some studies point to no significant difference between eradication rates guided by drug sensitivity testing and empirical treatment (Gomollón et al., 2000; Chen et al., 2019; Gingold-Belfer et al., 2021). For the above reasons, empirical treatment tends to dominate. Our study found that Ig Y-*H. pylori* improved the eradication rates of resistant strains after repeated treatment with antibiotic and had better clinical symptom relief and lower rates of adverse events.

Based on the characteristic of safety and no-resistance, Ig Y is more suitable for infants and children, immunodeficient patients (Rahman et al., 2013). In a randomized double-blind placebo-controlled trial in Japan, Takeuchi et al. (2016) used Ig Y to treat candida albicans infections in the elderly. Sarker et al. (2001) also reported encouraging results for Ig Y in alleviating diarrhea caused by rotavirus infection in children. The mechanisms for its safe and effective characteristic of Yolk antibodies may come from (a) specifically binding to antigenic epitopes of the pathogen and adsorbing the pathogen, (b) inhibiting the spread and multiplication of the pathogen, (c) enhancing the recognition and clearance of immune cells, and (d) neutralizing virulence factor (Rahman et al., 2013). Ig Y interferes with the colonization of *H. pylori*, promotes *H. pylori* excretion from the gastric mucosa, causes bacterial aggregation, and improves the recognition and phagocytosis efficiency of immune cells. In addition, Ig Y specifically recognizes and binds to virulence factors produced by *H. pylori* through antigen-antibody interactions, avoiding gastric mucosal damage and associated inflammatory responses (Nomura et al., 2005; Chalghoumi et al., 2009; Lee et al., 2021). As a candidate for passive immunization against pathogens, Ig Y is safer and more economical than antibiotics with high specificity and applicability.

There were some limitations to this study, firstly, it was a single-center clinical trial that requires further research in expanded population. Secondly, there was a bias in the subjective perceptions of patient during clinical symptom assessment. We used the Global Overall Symptom scale, a simple and validated scale (Heading et al., 2016), to record subjects’ pre- and post-treatment symptom scores using a 7-point scale. Finally, due to the experimental conditions, we did not use antibiotic susceptibility test or genotype resistance test to determine strain resistance. These limitations need to be further validated in a large sample, randomized controlled double-blind trial.

## Conclusion

In conclusion, for patients with repeated *H. pylori* eradication, Ig Y-*H. pylori* combined with bismuth-based quadruple therapy showed comparable efficacy to bismuth-based quadruple therapy with better symptomatic relief and fewer adverse effects. It can be recommended for the eradication of *H. pylori* infection.

## Data availability statement

The original contributions presented in this study are included in the article/Supplementary material, further inquiries can be directed to the corresponding author.

## Ethics statement

The studies involving human participants were reviewed and approved by the Institutional Review Board of Third Xiangya Hospital of Central South University. The patients/participants provided their written informed consent to participate in this study.

## Author contributions

CS, LJ, and XCX prepared the first draft of the manuscript. JC and HL wrote the study protocol. ZWF and LJH were investigators.
