# Supplementary material for: Egg yolk antibody combined with bismuth-based quadruple therapy in Helicobacter pylori infection rescue treatment: a single-center, randomized, controlled study
Source: Front Microbiol. 2023 May 16;14:1150129. doi: 10.3389/fmicb.2023.1150129 (PMC10225724; doi:10.3389/fmicb.2023.1150129)
Supplement: Supplementary file 1 [file Table_1.pdf]

Supplementary table1. Clinical Symptoms.

Symptoms	Description of symptoms
Upper abdominal pain	Pain in the upper abdomen just below the middle of the sternum
Bloating (or postprandial fullness)	An unpleasant sensation of persistent fullness in the stomach after eating
Upper abdominal discomfort	Other unexplained abdominal discomfort
Belching	Air escaping from the esophagus or stomach
Heartburn	Burning sensation that rises in the center of the chest down toward or into the neck
Nausea	Unpleasant sensations experienced in the upper abdomen or throat that urgently require vomiting
Acid reflux	Sour or bitter liquid flowing backward from the stomach into the esophagus
Early satiety	Feeling full shortly after starting to eat

Supplementary table2. The Global Overall Symptom (GOS) scale.

The Global Overall Symptom (GOS) scale
1. No problem
2. Minimal problem (can be easily ignored without effort)
3. Mild problem (can be ignored with effort)
4. Moderate problem (cannot be ignored but does not influence my daily activities)
5. Moderately severe problem (cannot be ignored and occasionally limits my daily activities)
6. Severe problem (cannot be ignored and often limits my concentration on daily activities)
7. Very severe problem (cannot be ignored and markedly limits my daily activities and often

Supplementary table3. Eradication rate.

Efficacy	Study group (n=50)	Control group (n=50)	*p*
Eradication success	42	40	
Eradication failure	7	10	
Case dropped	1	0	
Eradication rate			
ITT (95% CI)	84.0% (73.5%-94.5%)	80.0% (68.5%-91.5%)	0.603[a]
PP (95% CI)	85.7% (75.6%-95.9%)	80.0% (68.5%-91.5%)	0.451[a]

95% CI: 95% confidence interval. Eradication rate= number of successful eradications/number of eradications. [a] Chi-square test was used.

Supplementary table4. Eradication rates for different eradication cohorts.

Times of previous treatment	study group (n=49)	control group (n=50)	*p*
1	93.5% (29/31)	83.9% (26/31)	0.229[a]
⩾2	72.2% (13/18)	73.7% (14/19)	0.92[a]

[a] Chi-square test was used.

Supplementary table5. Clinical symptom relief after drug treatment.

Global Overall Symptom scale	study group (n=45)	control group (n=41)	*p*
Cure (n, %)	60.0% (27/45)	29.2% (12/41)	0.004[a]
Relief (n, %)	37.7% (17/45)	65.8% (27/41)	0.009[a]
Ineffective (n, %)	2.2% (1/45)	4.8% (2/41)	0.935[a]
p			0.017[a]

[a] Chi-square test was used.

Supplementary table 6. Adverse Events.

Adverse events	study group (n=49)	control group (n=50)
Allergies	0	1
Abdominal pain	1	0
Abdominal distention	0	1
Abdominal discomfort	0	2
Nausea	0	1
Diarrhea	2	0
Constipation	1	1
Overall	4 (8%)	6 (12%)
